# Pathogen communities of songbird-derived ticks in Europe’s low countries

**DOI:** 10.1186/s13071-017-2423-y

**Published:** 2017-10-18

**Authors:** Dieter Heylen, Manoj Fonville, Arieke Docters van Leeuwen, Arjan Stroo, Martin Duisterwinkel, Sip van Wieren, Maria Diuk-Wasser, Arnout de Bruin, Hein Sprong

**Affiliations:** 1 Evolutionary Ecology Group, University of Antwerp, Antwerp, Belgium; 2 Centre for Zoonoses & Environmental Microbiology, Centre for Infectious Disease Control, National Institute for Public Health and the Environment, Bilthoven, The Netherlands; 3 Centre for Monitoring of Vectors, Netherlands Food and Consumer Product Safety Authority, Ministry of Economic Affairs, Wageningen, The Netherlands; 4 Independent agricultural entrepreneur, Groningen, The Netherlands; 5 Resource Ecology Group, Wageningen University, Wageningen, The Netherlands; 6 EcoEpidemiology Lab, Columbia University, New York, USA

**Keywords:** Co-infection, Bird, *Ixodes ricinus*, *Borrelia burgdorferi* (*s.l.*), *Borrelia miyamotoi*, Rickettsiales

## Abstract

**Background:**

Birds play a major role in the maintenance of enzootic cycles of pathogens transmitted by ticks. Due to their mobility, they affect the spatial distribution and abundance of both ticks and pathogens. In the present study, we aim to identify members of a pathogen community [*Borrelia burgdorferi* (*s.l.*), *B. miyamotoi*, ‘*Ca.* Neoehrlichia mikurensis’, *Anaplasma phagocytophilum* and *Rickettsia helvetica*] in songbird-derived ticks from 11 locations in the Netherlands and Belgium (2012–2014).

**Results:**

Overall, 375 infested songbird individuals were captured, belonging to 35 species. Thrushes (*Turdus iliacus*, *T. merula* and *T. philomelos*) were trapped most often and had the highest mean infestation intensity for both *Ixodes ricinus* and *I. frontalis*. Of the 671 bird-derived ticks, 51% contained DNA of at least one pathogenic agent and 13% showed co-infections with two or more pathogens. *Borrelia burgdorferi* (*s.l.*) DNA was found in 34% of the ticks of which majority belong to so-called avian *Borrelia* species (distribution in *Borrelia*-infected ticks: 47% *B. garinii*, 34% *B. valaisiana*, 3% *B. turdi*), but also the mammal-associated *B. afzelii* (16%) was detected. The occurrence of *B. miyamotoi* was low (1%). Prevalence of *R. helvetica* in ticks was high (22%), while *A. phagocytophilum* and ‘*Ca.* N. mikurensis’ prevalences were 5% and 4%, respectively. The occurrence of *B. burgdorferi* (*s.l.*) was positively correlated with the occurrence of ‘*Ca.* N. mikurensis’, reflecting variation in susceptibility among birds and/or suggesting transmission facilitation due to interactions between pathogens.

**Conclusions:**

Our findings highlight the contribution of European songbirds to co-infections in tick individuals and consequently to the exposure of humans to multiple pathogens during a tick bite. Although poorly studied, exposure to and possibly also infection with multiple tick-borne pathogens in humans seems to be the rule rather than the exception.

## Background

Songbirds are swift transporters of ticks and tick-borne pathogens, spreading them over long distances on bird migration and dispersal routes. They are important pathogen reservoirs and carriers of infected ticks in areas that are less accessible to mammals, but still frequently visited by humans, such as islands, green space and gardens in urbanized areas [1–5]. Not only their contribution in the terrestrial cycles of pathogens has become clear during the past decades, but also their importance in maintaining tick populations is now generally recognized [6–9].

In Europe, bird-associated *Borrelia burgdorferi* (*s.l.*) species such as *B. garinii* and *B. valaisiana* [10–14] have been associated with human Lyme borreliosis [15, 16]. However, limited information is available on the birds’ contribution to the cycles of other human tick-borne pathogenic agents, as well as the mechanisms of co-occurrence of more than one pathogenic agent in individual birds and bird-derived ticks (“co-infection”) [17]. Understanding the mechanisms underlying co-infections in ticks is important, as co-infections in hosts in which tick bites are relatively low (e.g. humans) can result from the attachment of a single co-infected tick rather than sequential bites of multiple singly-infected ticks [18]. Simultaneous infections of multiple pathogen species can lead to increased pathogenicity, can affect pathogen proliferation dynamics in the hosts, can influence the host’s immune responses, can affect the distribution of pathogens in the host body and can complicate the diagnosis and treatment of disease [19–23].

Here, we investigated the (co-)occurrence of tick-borne-pathogens of humans and domesticated animals, for which songbirds are believed to potentially contribute to their maintenance, either as transmission facilitator (i.e. via local or systemic infections) or as vehicles of infected ticks. The pathogens considered are *B. burgdorferi* (*s.l.*) [4, 24], *Anaplasma phagocytophilum* [24–27], ‘*Candidatus* Neoehrlichia mikurensis’ [28, 29], *Rickettsia helvetica* [3, 17, 30, 31] and *Borrelia miyamotoi* [23, 29]. Among the avian *B. burgdorferi* (*s.l.*) species, *B. garinii* is responsible for human neuroborreliosis, while *B. valaisiana* has low pathogenicity, if any at all, for humans [32, 33]. The epidemiological importance for humans of *B. turdi* is currently unknown. *Borrelia miyamotoi* is a member of the relapsing fever group of *Borrelia* spirochaetes and can be hosted by rodents [34]*. Rickettsia helvetica* belongs to the spotted fever group and is an obligate intracellular bacterium, potentially causing cardiac and neurological problems in humans [35, 36]. *Anaplasma phagocytophilum* is an obligate intra-cellular rickettsia-like bacterium that can infect neutrophils causing granulocytic anaplasmosis in humans, livestock and companion animals [37]. The rickettsia-like bacterium ‘*Ca. *N. mikurensis’ is associated with febrile patients [38] and has been found in tissues of wild rodents [34, 39, 40].

The scope of our study is to identify the members of the pathogen community in tick species derived from songbird species in Europe’s Low Countries (Belgium and the Netherlands), to define their infection prevalence, and to investigate whether the occurrences of different pathogenic agents are independent of each other.

## Methods

### Bird trapping and collection of ticks

From 2012 to 2014, trained and experienced bird-banders opportunistically collected ticks from songbirds that were caught using Japanese mist nets in seven locations in the Netherlands (Eemshaven: 53°26′18.91″N, 6°50′7.77″E; Hijkerveld: 51°52′16.15″N, 4°28′49.84″E; Schiermonnikoog: 53°29′21.74″N, 6°13′51.27″E; Almere Oostvaardersdijk: 52°24′20.19″N, 5°10′40.45″E; Ankeveen: 52°15′51.19″N, 5°5′53.71″E; Nunspeet: 52°23′29.33″N, 5°49′15.12″E; and Oud Naarden: 52°18′16.93″N, 5°11′32.29″E) and four in Belgium (Merksplas: 51°21′29.48″N, 4°51′48.77″E; Vorselaar: 51°12′9.08″N, 4°46′15.17″E; Wilrijk: 51°10′5.91″N, 4°23′39.43″E; and Brecht: 51°20′58.75″N, 4°38′15.80″E). No information was obtained on the number of birds without ticks. Songbirds could be classified in nine categories following their foraging habitats (see Table 1) based on the information provided in a reference work [41] and the expert knowledge of two experienced bird-watchers of the University of Antwerp (J. Elst and D. Heylen). Immediately after collection, the ticks of an individual bird were immersed in a single tube filled with ethanol (80%), which was subsequently stored at -20 °C until species identification and DNA extraction. Ticks were identified to species and life stage by trained and experienced technicians who used various taxonomic keys [42–45].

**Table 1:** Numbers of *Ixodes ricinus* and *I. frontalis* tick stages (larvae, nymphs, adults) collected from songbirds

			*Ixodes ricinus*					*Ixodes frontalis*				
Scientific name (common name)	Foraging habitat^a^	Total infested birds	Larvae (Birds)	Nymphs (Birds)	Females (Birds)	Total ticks (Birds)	Mean infestation intensity	Larvae (Birds)	Nymphs (Birds)	Females (Birds)	Total ticks (Birds)	Mean infestation intensity
*Turdus merula* (Eurasian blackbird)	GF/GS	173	36 (24)	240 (122)		276 (139)	1.9	30 (30)	1 (1)	6 (5)	37 (36)	1.0
*Turdus philomelos* (Song thrush)	GF/GS	53	12 (12)	45 (40)		57 (51)	1.1	2 (1)	1 (1)	1 (1)	4 (3)	
*Turdus iliacus* (Redwing)	GF	33	22 (11)	52 (28)		74 (33)	2.2	8 (1)	2 (2)		10 (2)	
*Phoenicurus phoenicurus* (Common redstart)	OF	13	6 (6)	7 (7)		13 (13)	1.0					
*Prunella modularis* (Dunnock)	GF/GS	13	7 (3)	45 (12)		52 (13)	4.0					
*Erithacus rubecula* (European robin)	GF/GS	13	16 (7)	10 (8)		26 (13)	2.0					
*Troglodytes troglodytes* (Eurasian wren)	GF/GS	11	15 (7)	4 (4)		19 (11)	1.7					
*Sylvia communis* (Common whitethroat)	OB	8	3 (3)	13 (7)		16 (8)						
*Phylloscopus trochilus* (Willow warbler)	OF/OB	7	6 (2)	13 (6)		19 (7)						
*Sylvia curruca* (Lesser whitethroat)	GB/OB	5		6 (5)		6 (5)						
*Parus major* (Great tit)	OF/OS	5	2 (1)	3 (3)	1 (1)	6 (5)						
*Fringilla coelebs* (Common chaffinch)	GF/OF	4	2 (2)	1 (1)		3 (3)				1 (1)	1 (1)	
*Sylvia atricapilla* (Black cap)	OF/OB	4	4 (1)	4 (4)		8 (4)						
*Carduelis cabaret* (Lesser redpoll)	OF	4		3 (3)		3 (3)				1 (1)	1 (1)	
*Acrocephalus scirpaceus* (Eurasian reed warbler)	OW	3		4 (3)		4 (3)			1 (1)		1 (1)	
*Luscinia svecica* (Bluethroat)	GW/OW	2		8 (2)		8 (2)						
*Acrocephalus palustris* (Marsh warbler)	OW/OB	2		2 (2)		2 (2)						
*Passer domesticus* (House sparrow)	GU/GS	2	1 (1)	2 (1)		3 (2)						
*Passer montanus* (Eurasian tree sparrow)	GM/GS	2	1 (1)	1 (1)		2 (2)						
*Phylloscopus collybita* (Common chiffchaff)	OF	2		3 (2)		3 (2)						
*Sylvia borin* (Garden warbler)	OB	2^b^		1 (1)		1 (1)						
*Coccothraustes coccothraustes* (Hawfinch)	GF/OF	1		1 (1)		1 (1)						
*Ficedula hypoleuca* (Pied flycatcher)	OF	1		1 (1)		1 (1)						
*Garrulus glandarius* (Jay)	OF	1		1 (1)		1 (1)						
*Carduelis cannabina* (Linnet)	GM/OM	1		2 (1)		2 (1)						
*Poecile montanus* (Willow tit)	OF	1		1 (1)		1 (1)						
*Emberiza schoeniclus* (Reed bunting)	OW	1		1 (1)		1 (1)						
*Acrocephalus schoenabaenus* (Sedge warbler)	GW/OW	1		1 (1)		1 (1)						
*Alauda arvensis* (Eurasian skylark)	GM	1		1 (1)		1 (1)						
*Anthus pratensis* (Meadow pipit)	GM	1		1 (1)		1 (1)						
*Chloris chloris* (European greenfinch)	OF/OB	1								1 (1)	1 (1)	
*Turdus viscivorus* (Mistle thrush)	GF	1	1 (1)			1 (1)						
*Cyanistes caeruleus* (Blue tit)	OF	1								1 (1)	1 (1)	
*Carduelis spinus* (Eurasian siskin)	OF	1		1 (1)		1 (1)						
*Hippolais icterina* (Icterine warbler)	OB	1		1 (1)		1 (1)						
Total		375	134 (82)	479 (273)	1 (1)	614 (334)		40 (32)	5 (5)	11 (10)	56 (46)	

^a^Ground-foraging species include bird species that pre-dominantly feed on or close to the ground (< 0.5 m) of forests (GF), meadows (GM), bushes (GB), wetlands (GW), urban (GU), suburban (GS) habitat. Other species include species that pre-dominantly forage in higher vegetation strata (> 0.5 m) of forests (OF), bushes (OB) and wetlands (OW)

^b^For *Sylvia borin* (Garden warbler), we found one individual that was infested by a *Hyalomma* spp. nymph

### DNA extraction, qPCR assays and sequencing procedures

DNA was extracted from ticks using a Qiagen DNA extraction procedure. For the detection of *B. burgdorferi* (*s.l.*), a duplex qPCR was used, based on the detection of fragments of *ospA* and flagellin genes [5]. A conventional PCR assay, targeting the 5S-23S intergenic region (IGS), was performed for *B. burgdorferi* (*s.l.*) species identification [46]. Conventional PCR assays were carried out in a Px2 thermal cycler (Thermo Electron Corporation, Breda, the Netherlands) and visualized on a 2% agarose gel. Both strands of PCR products were sequenced by BaseClear (Leiden, the Netherlands), according to the company’s protocol and using the same forward and reverse primers as in conventional PCR. BLAST analyses and in-house molecular epidemiological databases (Bionumerics 7.1. - Applied Math, Belgium) were used to identify *Borrelia burgdorferi* (*s.l.*) species. These databases contain all our DNA sequences from (field) isolates, together with (reference) sequences from GenBank [47, 48].

For detection of *B. miyamotoi*, a qPCR assay was used that targets a region of the flagellin gene, specific for *B. miyamotoi* [49]. For detection of *A. phagocytophilum* and ‘*Ca.* N. mikurensis’ DNA, a single duplex qPCR assay was used, which is described elsewhere [50, 51]. This qPCR assay targets specific regions of genes *msp2* (major surface protein 2) for *A. phagocytophilum* and *groEL* (heat shock protein) for ‘*Ca.* N. mikurensis’. For detection of *R. helvetica*, we used a multiplex qPCR assay, targeting the *gltA* gene, as described earlier [52].

All qPCR runs were carried out in a final volume of 20 μl, containing IQ Multiplex Powermix (Bio-Rad, Hercules, CA, USA), 400 nM of primers and hydrolysis probes and 3 μl of DNA template. Conditions for PCR amplification were the following: 95 °C for 5 min, 60 cycles at 95 °C for 5 s and 60 °C for 35 s, followed by a final incubation step at 37 °C for 20 s. qPCR assays were carried out on a LightCycler 480 instrument (Roche Diagnostics Nederland B.V, Almere, the Netherlands) and analysis was performed by the instrument’s software (release 1.5.1.62). Quantification cycle (C_q_) values were calculated using the second derivative method.

### Statistical analysis

Generalized linear mixed effects models (GLMM) were fitted to test whether co-occurrences of different pathogen species in individual ticks were independent of each other (logit-link, binomial-distributed residuals), taking into account the correlation structure of co-feeding ticks that were obtained from the same individual [53]. In these models, individual birds were nested within bird species; both were modelled as random effects. For the inference of the maximum likelihood estimates of the fixed effects, Kenward Roger approximation was used to estimate the denominator degrees of freedom of the F-distributed test statistics, which takes into account the correlation of observations within the same cluster [53, 54]. For those bird species of which at least 10 individuals were caught, mean tick infestation intensity (i.e. the average number of ticks in infested individuals) was calculated. For bird species with at least 10 infected individuals per tick stage, estimates of proportions of infected ticks are given by their arithmetic mean ± standard error (i.e. the square root of the estimated variance/ the square root of the number of bird individuals) in the main text. Data management and statistical analyses were performed using SAS v9.2 (SAS Institute, Cary, North Carolina, USA).

## Results

### Tick infestations

Overall, 375 infested individual birds were trapped, belonging to 35 different species that could be classified into nine categories based on foraging habitats (Table 1). Thrushes (*Turdus merula*: 173, *T. philomelos*: 53, *T. iliacus*: 33) were trapped most often, representing 69% of the total number of infected birds. A total of 671 ticks was collected from the birds, belonging to three species: *Ixodes ricinus* (134 larvae, 479 nymphs and 1 adult female; collected from 334 birds), *I. frontalis* (40 larvae, 5 nymphs and 11 adult females; collected from 46 birds) and one *Hyalomma* spp. nymph from *Sylvia borin* (Table 1). Six individual birds (2 *T. merula*, 2 *T. iliacus*, 1 *T. philomelos* and 1 *Acrocephalus scirpaceus*) were infected with both *I. ricinus* and *I. frontalis*.

For *I. ricinus,* tick infestation intensity was highest for *Prunella modularis* (4.0 ± 1.5; *n* = 13), followed by *T. iliacus* (2.2 ± 0.4; *n* = 33), *Erithacus rubecula* (2.0 ± 0.6; *n* = 13) and *T. merula* (1.9 ± 0.2; *n* = 139). For *I. frontalis,* the infestation intensity for *T. merula* was 1.0 ± 0.0 (*n* = 36).

### Pathogens in bird-derived ticks

All 671 ticks were individually screened for the presence of *B. burgdorferi* (*s.l.*), *B. miyamotoi*, *A. phagocytophilum*, ‘*Ca.* N. mikurensis’ and *R. helvetica* (Table 2). Overall, 50.9% (341/670) of ixodid ticks collected were found infected with one or more of these pathogens [*I. ricinus*: 54% (333/614), *I. frontalis* 14% (8/56)]. We found none of these pathogens in the one *Hyalomma* spp. nymph.

**Table 2:** Numbers of infected songbird-derived *Ixodes ricinus* (Ir) and *I. frontalis* (If) stages

Scientific name	*B. burgdorferi* (*s.l.*)			*A. phagocytophilum*			‘*Ca.* N. mikurensis’		*R. helvetica*			*B. miyamotoi*	
	Larvae Ir (%)	Nymphs Ir (%)	Adults Ir/If (%)	Larvae Ir (%)	Nymphs Ir (%)	Adults Ir/If (%)	Larvae Ir (%)	Nymphs Ir (%)	Larvae Ir/If (%)	Nymphs Ir (%)	Adults Ir/If (%)	Larvae Ir (%)	Nymphs Ir (%)
*Turdus merula*	15 (30.5)	125 (53.6)	−/4	1 (4.2)	20 (7.6)	−/2		9 (5.3)	4 (14.6)/1 (3.3)	53 (24.2)			1 (9.1 10^-4^)
*Turdus philomelos*	11 (91.6)	26 (61.7)			2 (5)			9 (22.5)	3 (25.0)	13 (29.2)	−/1		1 (1.3 10^-2^)
*Turdus iliacus*	3 (9.1)	9 (25.4)		3 (6.8)	3 (8.0)		1 (4.5)	3 (1.8)	3 (15.9)	5 (10.7)			
*Phoenicurus phoenicurus*		4						1	1	2			
*Prunella modularis*		11 (20.2)			2 (1.4)			2 (2.9)	1	9 (23.4)			
*Erithacus rubecula*	1	1							10	4		1	
*Troglodytes troglodytes*	2						1		4	1			
*Sylvia communis*		1						2	1	6			
*Phylloscopus trochilus*		5							1	4			
*Sylvia curruca*		1								2			
*Parus major*									2	1	1/−		
*Fringilla coelebs*													
*Sylvia atricapilla*								1	1	2			
*Carduelis cabaret*		1	−/1							2			
*Acrocephalus scirpaceus*										1			
*Luscinia svecica*													
*Acrocephalus palustris*													
*Passer domesticus*		1							1				
*Passer montanus*	1	1											
*Phylloscopus collybita*								1		1			1
*Sylvia borin*		1											
*Coccothraustes coccthraustes*										1			
*Ficedula hypoleuca*		1											
*Garrulus glandarius*													
*Carduelis cannabina*													
*Poecile montanus*										1			
*Emberiza schoeniclus*													
*Acrocephalus schoenabaenus*													
*Alauda arvensis*					1					1			
*Anthus pratensis*													
*Chloris chloris*													
*Turdus viscivorus*	1												
*Cyanistes caeruleus*											−/1		
*Carduelis spinus*													
*Hippolais icterina*													
Total per stage	34	188	−/5	4	28	−/2	2	28	32/1	109	1/2	1	3
*I. ricinus* infected/total (%)	222/614 (36.2)			32/614 (5.2)			30/614 (4.9)		142/614 (23.1)			4/614 (0.065)	
*I. frontalis* infected/total (%)	5/56 (8.9)			2/56 (3.6)			0/56 (0)		3/56 (5.3)			0/56 (0)	

We detected *B. burgdorferi* (*s.l.*) DNA in 33.9% (227/670) of all ixodid ticks. The highest proportion of *B. burgdorferi* (*s.l.*) positive larvae was observed in *T. philomelos* (91.6 ± 0.8%; *n* = 12 infested birds)*,* followed by *T. merula* (30.5 ± 9.2%; *n* = 24 birds) and *T. iliacus* (9.09 ± 9.09%; *n* = 11 birds). The proportions of positive *I. ricinus* were higher in nymphs than in larvae when collected from *T. merula* (53.6 ± 4.2%; *n* = 122 birds) and *T. iliacus* (25.4 ± 8.3%; *n* = 28 birds) but not from *T. philomelos* (61.7 ± 7.7%; *n* = 40 birds). From latter members of the Turdidae family, we mainly found avian species (*B. garinii*, *B. valaisiana* and *B. turdi*) in both larvae and nymphs (Table 3). In *Prunella modularis* (mean prevalence: 20.2 ± 8.5%; *n* = 12 infested birds) only the mammal-associated *B. afzelii* (8 infected nymphs belonging to 5 infested birds) was found. Overall, for the complete set of *Borrelia*-infected ticks for which the *Borrelia*-genotyping was successful (173 tick individuals belonging to 15 bird species), avian species were detected in all developmental stages, while *B. afzelii* was detected in nymphs only (Table 3). *Borrelia turdi* was found in *I. frontalis* (2 adult females) and *I. ricinus* (3 nymphs).

**Table 3:** *Borrelia burgdorferi* (*s.l.*) species in *Ixodes ricinus* and *I. frontalis* collected from 110 bird individuals

Scientific name	*B. garinii*			*B. valaisiana*		*B. afzelii*		*B. turdi*			Total infested birds
	Larvae Ir	Nymphs Ir	Adults If	Larvae Ir	Nymphs Ir	Larvae Ir	Nymphs Ir	Larvae Ir	Nymphs Ir	Adults If	
*Turdus merula*	5	47	1	5	44		4		2	1	64
*Turdus philomelos*	2	7		1	4		5		1		20
*Prunella modularis*							8				5
*Turdus iliacus*	3	5					1				5
*Phoenicurus phoenicurus*		2					1				3
*Phylloscopus trochilus*							3				2
*Troglodytes troglodytes*	2										2
*Carduelis cabaret*							1			1	2
*Ficedula hypoleuca*		1									1
*Sylvia curruca*							1				1
*Sylvia communis*							1				1
*Passer domesticus*		1									1
*Passer montanus*				1							1
*Erithacus rubecula*							1				1
*Turdus viscivorus*	1										1
Total per stage	13	63	1	7	48		26		3	2	
Infected/total (%)	77/163 (47.2)			55/163 (33.7)		26/163 (15.9)		5/163 (3.1)			

The occurrence of *B. miyamotoi* in ticks was very low [prevalence in ixodid ticks: 0.6% (4/670)]. It was only found in one *I. ricinus* larva from *E. rubecula* and one *I. ricinus* nymphs from *T. merula*, *Phylloscopus collybita*, *T. philomelos* each.

For *A. phagocytophilum*, we detected DNA in 5.1% (34/670) of all ixodid ticks. The nymphal infection prevalence in the four bird species with more than 10 infested birds varied between 1.4 ± 0.9% (*P. modularis*) and 8 ± 4.9% (*T. iliacus*). Furthermore, *A. phagocytophilum* DNA was found in two *I. frontalis* females from one individual blackbird as well.

The overall prevalence of ‘*Ca.* N. mikurensis’ in the ixodid ticks was 4.4% (30/670). A high prevalence was found in *I. ricinus* nymphs collected from *T. philomelos* (22.5 ± 6.7%; *n* = 40 infested birds), but below 5.5% in the other Turdidae. Only two larvae, *I. ricinus* collected from *Troglodytes troglodytes* and *T. iliacus*, carried ‘*Ca.* N. mikurensis’ DNA*.*

Compared to the other rickettsial infections, the number of ticks with *R. helvetica* - DNA was high [overall prevalence: 21.6% (145/670); Table 2]. Infection prevalence ranged from 10.7 ± 5.4% (*T. iliacus*; *n* = 28 infested birds) to 29.2 ± 7.2% (*T. philomelos*; *n* = 40 infested birds) in those bird species with at least 10 nymphs. But also in the bird species of which we obtained a smaller amount of information (i.e. less than 10 infested birds) high prevalence was registered (e.g. nymphs in *E. rubecula*: 41.7 ± 17.5%; *n* = 8; *Sylvia communis*: 42.9 ± 20.2%; *n* = 7; *Phoenicurus phoenicurus*: 28.6 ± 18.4%; *n* = 7). *Rickettsia helvetica*-positive *I. ricinus* larvae were collected from 12 different songbird species. In general, larval prevalence was lower, but still high (range in Turdidae: 14.6 ± 7.0% – 25.0 ± 13.1%). In addition, this bacterium was detected in an *I. frontalis* larva collected from *T. merula* and two *I. frontalis* adult females from *C. caeruleus* and *T. philomelos*.

### Co-infection

Over 10% (13.4%, 90/671) of bird-derived ticks contained DNA of more than one pathogenic agent. At the bird level, 19.7% of individual birds (74/375) carried ticks with a co-infection. In *I. ricinus* larvae and nymphs, the most common pathogen combinations were ‘*B. burgdorferi* (*s.l.*) + *R. helvetica*’ (larvae: 6 ticks; nymphs: 46 ticks over 44 birds), followed by ‘*B. burgdorferi* (*s.l.*) + ‘*Ca.* N. mikurensis’ (larvae: 0 ticks; nymphs: 17 ticks over 17 birds) and ‘*B. burgdorferi* (*s.l.*) + *A. phagocytophilum*’ (larvae: 0 ticks; nymphs: 17 ticks over 11 birds).

There was no statistical evidence for an association between *B. burgdorferi* (*s.l.*) and *R. helvetica* neither in *I. ricinus* nymphs (*F*_(1,204)_ = 0.35, *P* = 0.55) nor *I. ricinus* larvae (*F*_(1,21)_ = 0.04, *P* = 0.83) when taking host species and bird individual into account as a random effects. The same holds for *B. burgdorferi* (*s.l.*) and *A. phagocytophilum* (nymphs: *F*_(1,205)_ = 3.00, *P* = 0.09; larvae: no model convergence). We did find a positive correlation between the occurrence of ‘*Ca.* N. mikurensis’ and *B. burgdorferi* (*s.l.*) (nymphs: logit _absence-presence_ = -1.02 ± 0.46, *F*_(1,205)_ = 4.9, *P* = 0.028; larvae: no model convergence). The analysis on the level of individual birds in which for each bird a binary value (“1”: at least one infected tick, or “0”: no infected ticks) was assigned as response variable did not change the conclusions (no significant association between *B. burgdorferi* (*s.l.*) and *A. phagocytophilum*, nor *B. burgdorferi* (*s.l.*) and *R. helvetica*: all *P*-values > 0.33). The same holds for the association between ‘*Ca.* N. mikurensis’ and *B. burgdorferi* (*s.l.*) (logit _absence-presence_ = -1.48 ± 0.49, *F*_(1,300)_ = 8.99, *P* = 0.003). Due to low sample sizes, associations between other pathogen combinations could not be analyzed.

The following rare combinations of pathogens in ticks were found: *R. helvetica* + ‘*Ca.* N. mikurensis’ (larvae: 1 tick; nymphs: 9 ticks over 9 birds); *A. phagocytophilum* + *R. helvetica* (larvae: 1 tick; nymphs: 7 ticks over 6 birds); *B. burgdorferi* (*s.l.*) + *B. miyamotoi* (1 nymph); *B. miyamotoi* + ‘*Ca.* N. mikurensis’ (1 nymph). Six nymphs (over 6 birds) carried DNA of *B. burgdorferi* (*s.l.*), *R. helvetica* and ‘*Ca.* N. mikurensis’ and three nymphs were infected with the combination *B. burgdorferi* (*s.l.*) + *R. helvetica* + *A. phagocytophilum* (over 2 birds). Also in the smaller set of *I. frontalis* ticks, we found the co-infection *B. burgdorferi* (*s.l.*) + *A. phagocytophilum* in two adult females collected from a single *T. merula*.

## Discussion

We have shown that half of the songbird-derived *I. ricinus* ticks, that readily feed on humans, contained DNA of one or more bacteria that are pathogenic to humans: *B. burgdorferi* (*s.l.*), *R. helvetica*, *A. phagocytophylum*, *B. miyamotoi*, ‘*Ca.* N. mikurensis’. The presence of the DNA in the ticks shows that songbirds carry infected ticks and may facilitate bacterium transmission. Transmission facilitation via birds may occur either via the infection of bird tissue on which ticks feed or via co-feeding of ticks in close spatial and temporal proximity to each other. The latter transmission pathway can occur in the absence of a systemic infection, allowing some pathogens (e.g. *Borrelia* species) to evade the hostile immune system of otherwise incompetent hosts [55].

Ground-dwelling birds, especially the members of the family Turdidae, had the highest infestation intensities and also yielded the highest numbers of infected ticks overall. They are known to run a greater risk of *I. ricinus* exposures, as they forage inside the habitat of this abundant tick species (i.e. ground and lower vegetation strata) [4, 56]. Particularly the blackbird (*T. merula*) and the song thrush (*T. philomelos*), two very common birds of European woodlands and gardens, contributed strongly to the number of infected ticks. In line with other European studies, birds were predominantly infested by immature *I. ricinus* stages and rarely by adult females. Adult *I. ricinus* are typically found on medium-sized and larger mammals (e.g. roe deer) on which they copulate [57]. In contrast but not surprising, we found substantial numbers of semi-engorged adult female *I. frontalis* on the birds; all developmental stages *I. frontalis* feed on birds [42].

We found a strong association of *B. garinii*, *B. valaisiana* and *B. turdi* with avian reservoir hosts, which has previously been shown by numerous European field studies concluding that birds act both as competent reservoirs and transmitters for these species [10–12, 29, 58–60]. Given that vertical transmission of *B. burgdorferi* (*s.l.*) spirochetes in *I. ricinus* ticks only rarely occurs [61, 62], their presence in larvae (Tables 2, 3) indicates that they were acquired either via (local) infection in the host or via co-feeding with an infected nymph. *Borrelia turdi*, recently discovered in Europe and strongly associated with *I. frontalis*, was also found in *I. ricinus* nymphs. Transmission experiments have shown that *I. ricinus* can transmit *B. turdi* to naïve avian hosts and, seen the extreme host range of this tick species, *I. ricinus* could potentially act as a bridging vector towards mammals, including human [60]. Recent experimental and observational studies based on larval and nymphal infections show non-homogeneous distributions of the avian *Borrelia* species in bird-derived ticks, indicating differential transmission and amplification of these species depending on the avian reservoir hosts and tick species [12, 60, 63].

An interesting outcome of our study and previous field studies is that several of the ground-dwelling birds (*T. merula*, *T. philomelos*, *E. rubecula* and *P. modularis*) were frequently infested with ticks that carried *B. afzelii*, a species that is associated with rodent reservoir hosts [64, 65]. The fact that all *B. afzelii*-infected ticks were nymphs suggests that these individuals had fed as a larva on a *B. afzelii*-infected mammal, moulted and maintained their infections when feeding on the birds. Findings of bird-derived infected larvae in other studies have led to speculations that particular strains of *B. afzelii* can also use bird hosts for transmission [14]. PCR-based screening outcomes, like ours and others, should, however, be interpreted with caution as they are based on the detection of specific DNA sequences and do not necessarily mean that viable, infectious microorganisms are present. A recent experimental study investigating transmission of *B. afzelii* in blackbirds and great tits showed that nymph-to-adult transstadial transmission of *B. afzelii* DNA could occur. However, the positive signal in the adult ticks turned out not to be viable and infectious spirochetes, as shown by the BSK culture test [66]. It is, therefore, necessary to identify the *B. afzelii* strains found in bird-derived ticks from the wild and test via culture-based infection methods and tick transmission experiments whether they are still infectious and transmittable after being exposed to bird blood during tick feeding.

Also for the more sporadically observed mammal-associated pathogens (*B. miyamotoi* and ‘*Ca.* N. mikurensis’) that we detected in bird-derived ticks, including larvae (Table 2), experiments are needed to investigate whether viable bacteria survive the birds immune system and/or are transmitted during tick feeding. Studies in the USA and Europe have implicated small rodent species as the reservoir hosts for *B. miyamotoi* [34, 67], but a limited number of studies reported *B. miyamotoi* infections from bird-derived ticks as well [17, 29, 68]. For the rodent-associated ‘*Ca.* N. mikurensis’ [50] observed in bird-derived *I. ricinus* larvae and nymphs of our study and others [28, 29], a role for songbirds as transmission facilitators could be expected.

Compared to the other *A. phagocytophilum* and ‘*Ca.* N. mikurensis’, the number of ticks with *R. helvetica* DNA was high. A good comprehension of the transmission dynamics of rickettsial bacteria in songbirds is still lacking. Within infected ticks, a proportion of the bacteria could have a maternal origin, as *R. helvetica*, in contrast to *Borrelia burgdorferi* (*s.l.*), can be transmitted transovarially [69, 70]. However, the experimental study of Heylen et al. [17] using great tits (*Parus major*) exposed to a community of pathogens, clearly shows rapid *R. helvetica* transmission via co-feeding (cf. mammals [71, 72]) and/or fast systemic infection (as found in mammals experimentally injected with different rickettsial species [73]). Our and other’s finding of *R. helvetica* in bird-derived ticks, including larvae [3, 29–31, 51, 74, 75] reinforces the presumption that songbirds can become bacteraemic and effectively facilitate the transmission of this pathogen via host tissue [30].

Further, our study provides evidence that ground-dwelling birds, especially thrushes, are important hosts in the transmission cycles of *A. phagocytophilum*. Bacteraemia of this pathogen has been shown to develop in songbirds [30], which is likely the reason for the reports of infected bird-derived ticks here and other locations [25, 26, 29, 30, 76]. Probably not all birds are equally competent in the transmission; in a great tit (*Parus major*) experiment by Heylen et al. [17] no transmission facilitation occurred despite the presence of *A. phagocytophilum* in challenge nymphs. Our finding of infections in a bird-specialized tick (*I. frontalis*) that is never found on other vertebrate hosts (two infected adult females co-feeding with infected *I. ricinus* nymphs on the same blackbird individuals) gives further indication that birds facilitate *A. phagocytophilum* transmission, either via co-feeding transmission or systemic infections. Although the host-specific strains of *A. phagocytophilum* were not identified, the avian ecotype IV that has been isolated by Jahfari et al. [51] from blackbird tissues and blackbird-derived ticks is to be expected.

The co-infections found in individual ticks and birds strongly suggest that simultaneous transmission of different bacterium species can occur and that birds are permissive for multiple pathogens, as experimentally shown in Heylen et al. [28]. Especially, the fact that co-infections were found in (sets of) larvae provides the strongest indication. However, larvae could also acquire pathogens via the maternal line from other hosts than the individual songbird from which they were collected, through vertical transmission (e.g. in *B. miyomatoi* and *R. helvetica*) [69, 70, 77]. In larvae, the most frequent observed co-infection was *B. burgdorferi* (*s.l.*) with *R. helvetica* (*E. rubecula*, *T. troglodytes*, *T. philomelos*), but also *A. phagocytophilum* with *R. helvetica* and ‘*Ca.* N. mikurensis’ with *R. helvetica* (both from *T. iliacus*) were observed*.* Only for one bacterial combination, ‘*Ca.* N. mikurensis’ + *B. burgdorferi* (*s.l.*), we found that the occurrence of the one pathogen is more likely when another pathogenic agent is present. Interestingly, also in mammals, this combination of pathogens was much higher than expected from the prevalence of each pathogen [39]. This positive association could be the result of variation in general susceptibility among birds, but could also indicate transmission facilitation, as has been suggested in other studies on tick-borne co-infections [18, 34, 78]. The pathways that lead to facilitation can only be elucidated with experimental studies in which pathogen-driven physiological, cellular and biochemical interactions are disentangled.

## Conclusions

Our findings highlight the contribution of songbirds to co-infections in individual ticks. In addition, not only avian but also mammalian bacterium species are transported via bird-derived ticks, highlighting the need to experimentally test whether latter pathogens are viable and infectious in birds. Furthermore, future studies should focus on the reservoir competence of members in the bird community and how the different vector-bird-niches contribute to the pathogen transmission dynamics.
